# A drug-resistant β-lactamase variant changes the conformation of its active-site proton shuttle to alter substrate specificity and inhibitor potency

**DOI:** 10.1074/jbc.RA120.016103

**Published:** 2025-01-13

**Authors:** Victoria Soeung, Shuo Lu, Liya Hu, Allison Judge, Banumathi Sankaran, B. V. Venkataram Prasad, Timothy Palzkill

**Affiliations:** 1 Department of Pharmacology and Chemical Biology, Baylor College of Medicine, Houston, Texas, USA; 2 Department of Biochemistry and Molecular Biology, Baylor College of Medicine, Houston, Texas, USA; 3 Department of Molecular Virology and Microbiology, Baylor College of Medicine, Houston, Texas, USA; 4 Department of Molecular Biophysics and Integrated Bioimaging, Berkeley Center for Structural Biology, Lawrence Berkeley National Laboratory, Berkeley, California, USA

**Keywords:** beta-lactamase, antibiotics, antibiotic resistance, enzyme inhibitor, enzyme structure, enzyme kinetics

## Abstract

Lys^234^ is one of the residues present in class A β-lactamases that is under selective pressure due to antibiotic use. Located adjacent to proton shuttle residue Ser^130^, it is suggested to play a role in proton transfer during catalysis of the antibiotics. The mechanism underpinning how substitutions in this position modulate inhibitor efficiency and substrate specificity leading to drug resistance is unclear. The K234R substitution identified in several inhibitor-resistant β-lactamase variants is associated with decreased potency of the inhibitor clavulanic acid, which is used in combination with amoxicillin to overcome β-lactamase–mediated antibiotic resistance. Here we show that for CTX-M-14 β-lactamase, whereas Lys^234^ is required for hydrolysis of cephalosporins such as cefotaxime, either lysine or arginine is sufficient for hydrolysis of ampicillin. Further, by determining the acylation and deacylation rates for cefotaxime hydrolysis, we show that both rates are fast, and neither is rate-limiting. The K234R substitution causes a 1500-fold decrease in the cefotaxime acylation rate but a 5-fold increase in *k*_cat_ for ampicillin, suggesting that the K234R enzyme is a good penicillinase but a poor cephalosporinase due to slow acylation. Structural results suggest that the slow acylation by the K234R enzyme is due to a conformational change in Ser^130^, and this change also leads to decreased inhibition potency of clavulanic acid. Because other inhibitor resistance mutations also act through changes at Ser^130^ and such changes drastically reduce cephalosporin but not penicillin hydrolysis, we suggest that clavulanic acid paired with an oxyimino-cephalosporin rather than penicillin would impede the evolution of resistance.

β-Lactam antibiotics are among the most often used agents for treatment of bacterial infections. Unfortunately, because of their widespread use, bacterial resistance to these drugs now represents a threat to antimicrobial therapy. The major mechanism of resistance to β-lactam antibiotics is the bacterial production of β-lactamases, which hydrolyze the amide bond of the β-lactam ring of these drugs to render them ineffective. β-Lactamases are grouped into four classes, A, B, C, and D, based on amino acid sequence homology (1). Enzymes from classes A, C, and D are serine hydrolases that act through a covalent, acyl-enzyme intermediate that is subsequently hydrolyzed by an activated water (2). Enzymes from class B are zinc metalloenzymes where the β-lactam is hydrolyzed through direct attack of an activated water or hydroxide (3, 4, 5).

Serine active-site β-lactamases such as the common class A enzymes hydrolyze the amide bond in β-lactam antibiotics through sequential acylation and deacylation steps (Fig. 1). In class A enzymes, the catalytic Ser^70^ residue attacks the carbonyl carbon of the β-lactam, resulting in cleavage of the amide bond to form a covalent acyl-enzyme intermediate (6, 7). Acylation is facilitated by the transfer of a proton from Ser^130^, via a proton shuttle from Lys^73^, to the leaving group nitrogen of the amide (8, 9) (Fig. 1). The deacylation reaction is catalyzed by the active-site residue Glu^166^, which serves as a general base to activate a catalytic water molecule for attack on the carbonyl carbon of the acyl-intermediate and subsequent formation of the hydrolyzed product. A minimal kinetic scheme for β-lactamase–catalyzed hydrolysis of β-lactams is shown in Fig. 2*A*. These active-site residues are conserved across all class A β-lactamases, consistent with their key role in catalysis.

**Figure 1:**
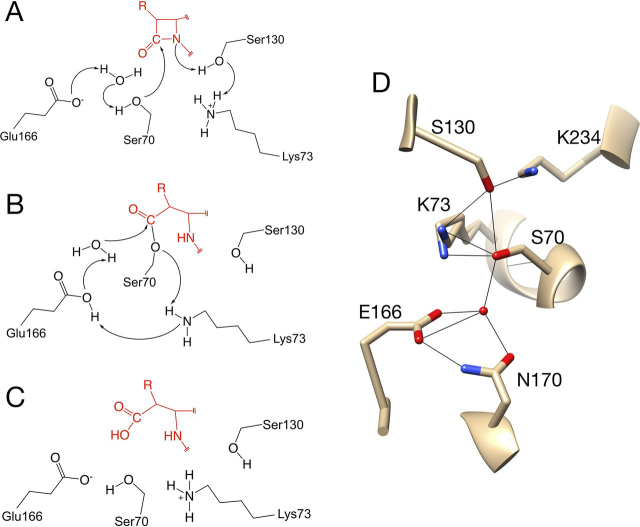
**Serine** β**-lactamase mechanism and key catalytic residues.***A*, the Ser^70^ nucleophile is activated by a base, here shown as Glu^166^ via a catalytic water. During cleavage of the amide bond, the leaving group nitrogen is protonated by Ser^130^ via a proton shuttle from Lys^73^. The result is the formation of the acyl-enzyme intermediate. Note that the ring fused to the β-lactam ring is truncated for clarity as indicated by *break lines* (//). *B*, the carbonyl carbon of the acyl-enzyme intermediate is attacked by the catalytic water, which is activated by Glu^166^ serving as a general base. Glu^166^ is activated by Lys^73^. The leaving group oxygen on Ser^70^ is protonated by Lys^73^. *C*, the hydrolyzed β-lactam product is released to regenerate the enzyme. *D*, structure of the key active-site residues of CTX-M-14 β-lactamase (PDB entry 1YLT) described in *A–C*. Note that residue Lys^234^ forms a hydrogen bond with Ser^130^ to lower its p*K_a_* to facilitate donating a proton to the leaving group nitrogen of the β-lactam. The two conformations of Lys^73^ are proposed to reflect states relevant to the proton shuttle role of this residue as depicted in *A* and *B*.

**Figure 2:**
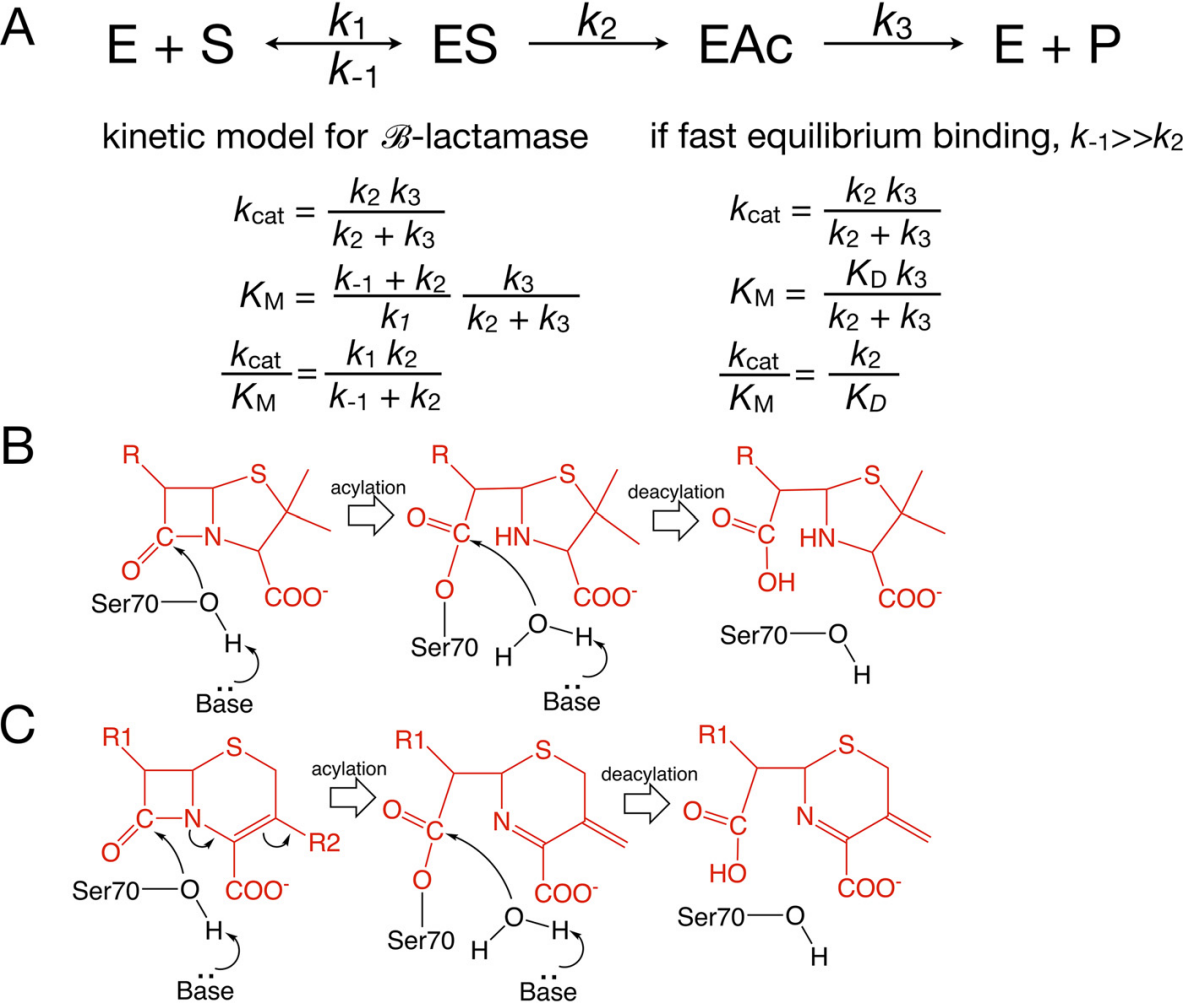
**Kinetic model for serine active-site β-lactamases.***A*, at the *top* is the minimal scheme for the reaction with *E* as free enzyme, *S* as substrate, *ES* as enzyme-substrate complex, *EAc* as acyl-enzyme intermediate, and *P* as product. Also shown are the equations for *k*_cat_, *K_m_*, and *k*_cat_/*K_m_* based on the kinetic scheme. A simplified model is shown at the *right* based on the assumption that *k*_−1_ ⋙ *k*_2_ (*i.e.* that the substrate-binding reaction is in rapid equilibrium). *B*, schematic illustration of the β-lactamase mechanism for penicillin hydrolysis with Ser^70^ serving as the nucleophile for attack on the β-lactam carbonyl carbon to form the acyl-enzyme intermediate. Subsequently, Glu-166 acts as a base to activate water for attack on the carbonyl carbon of the acyl-enzyme intermediate, leading to hydrolyzed product. *C*, schematic illustration of the mechanism for cephalosporin hydrolysis. Note that the R2 group is eliminated. The elimination of the R2 group is shown coincident with cleavage of the β-lactam amide bond, although elimination can also occur after cleavage of the amide.

In response to increasing antibiotic resistance due to the production of β-lactamases, extended-spectrum oxyimino-cephalosporins were introduced in the 1980s. These antibiotics are poor substrates for widespread class A β-lactamases, such as TEM-1 and SHV-1, and therefore bacteria containing these enzymes remain susceptible to the drugs. The use of oxyimino-cephalosporins, however, led to selective pressure for the evolution of resistance, resulting in mutations in TEM and SHV that expand their substrate specificity (10).

β-Lactamase inhibitors have also been developed to combat antibiotic resistance (11). Clavulanic acid, tazobactam, and sulbactam are covalent inhibitors of many class A β-lactamases. They act through secondary reactions that occur after acylation due to secondary ring opening and fragmentation that attaches various fragments to Ser^70^ as well as one product that is cross-linked to Ser^130^ (11). In response to the selective pressure of inhibitor use, several inhibitor-resistant variants with mutations in TEM-1 and SHV-1 have been identified. These include the substitutions S130G, M69I, M69V, K234R, and R244S (12, 13, 14, 15, 16). More recently, the diazabicyclooctane, avibactam, has been introduced, which is also a covalent inhibitor that cycles between acylation and deacylation without secondary ring fragmentation (17, 18).

The selective pressure of oxyimino-cephalosporin use has also led to the emergence of a new family of β-lactamases that readily hydrolyze these drugs. The CTX-M family first appeared in the late 1980s and now represents the most widespread extended-spectrum β-lactamases in Gram-negative bacteria. The CTX-M β-lactamases are class A enzymes that have a broad substrate specificity that includes penicillins and cephalosporins, including the oxyimino-cephalosporin cefotaxime (10). They are divided into five clusters based on amino acid sequence homology, including CTX-M-1, CTX-M-2, CTX-M-8, CTX-M-9, and CTX-M-25, with the names based on the prominent member of each subgroup (19). The subgroups differ from one another by ≥10% amino acid sequence divergence, and each subgroup contains variants that differ by ≤5% sequence divergence (19).

The substrate specificity of β-lactamases is a key determinant of antibiotic resistance in that it determines what drugs are hydrolyzed and therefore the susceptibility of bacteria harboring the enzymes. Most class A β-lactamases efficiently hydrolyze penicillins and cephalosporins to provide bacterial resistance to these drugs. For example, the common TEM-1 β-lactamase hydrolyzes benzylpenicillin with a catalytic efficiency in the range of 10^7^–10^8^m^−1^ s^−1^ and the older cephalosporin, cephalothin, with a *k*_cat_/*K_m_* of 3 × 10^5^m^−1^ s^−1^, whereas it poorly hydrolyzes the oxyimino-cephalosporin cefotaxime, with a *k*_cat_/*K_m_* of 2 × 10^3^m^−1^ s^−1^ (10, 20). In contrast, CTX-M β-lactamases hydrolyze benzylpenicillin with a *k*_cat_/*K_m_* of 5 × 10^6^m^−1^ s^−1^, cephalothin with a *k*_cat_/*K_m_* of 2 × 10^7^m^−1^ s^−1^, and cefotaxime with a *k*_cat_/*K_m_* of 3 × 10^6^m^−1^ s^−1^ (10, 21). Thus, both TEM-1 and CTX-M enzymes efficiently catalyze the hydrolysis of penicillins and early-generation cephalosporins, but CTX-M also hydrolyzes the newer, oxyimino-cephalosporin cefotaxime ∼1,000-fold faster than the TEM-1 enzyme (10). This enhanced hydrolysis of cefotaxime provides resistance to bacteria containing CTX-M enzymes and has led to the rapid spread of CTX-M–encoding plasmids among pathogens (19).

Another active-site residue that is conserved across most class A enzymes and among the serine β-lactamases in general is Lys^234^ (Fig. 1*D*). Based on structures of β-lactamases in complex with β-lactam antibiotics, Lys^234^ has been suggested to facilitate substrate binding by providing an electrostatic environment favorable for interaction with the C3/C4 carboxylate group found in all of the β-lactams (6, 22, 23) (Fig. 3). In addition, Lys^234^ is located near the proton shuttle residue, Ser^130^, and may act to lower the p*K_a_* of the serine hydroxyl to facilitate proton transfer to the nitrogen leaving group of the β-lactam during the acylation reaction (8, 9) (Fig. 1). Studies with TEM-1 β-lactamase have shown that a K234T substitution that removes the positive charge results in greatly reduced (∼50-fold) *k*_cat_ values and 50-fold increased *K_m_* values for benzylpenicillin hydrolysis, suggesting that Lys^234^ is important for both substrate binding and catalysis (24). In addition, the K234T substitution essentially eliminates cephalosporin hydrolysis by the TEM-1 enzyme (24). In contrast, a K234R substitution in TEM-1 has relatively modest effects on penicillin hydrolysis (*k*_cat_ reduced 2-fold) and no effect on the rate of cephalosporin hydrolysis (24). Taken together, these results suggest that Lys^234^ may contribute to substrate binding, through interaction with the C3/C4 carboxylate group, and to catalysis, by lowering the p*K_a_* of Ser^130^.

**Figure 3:**
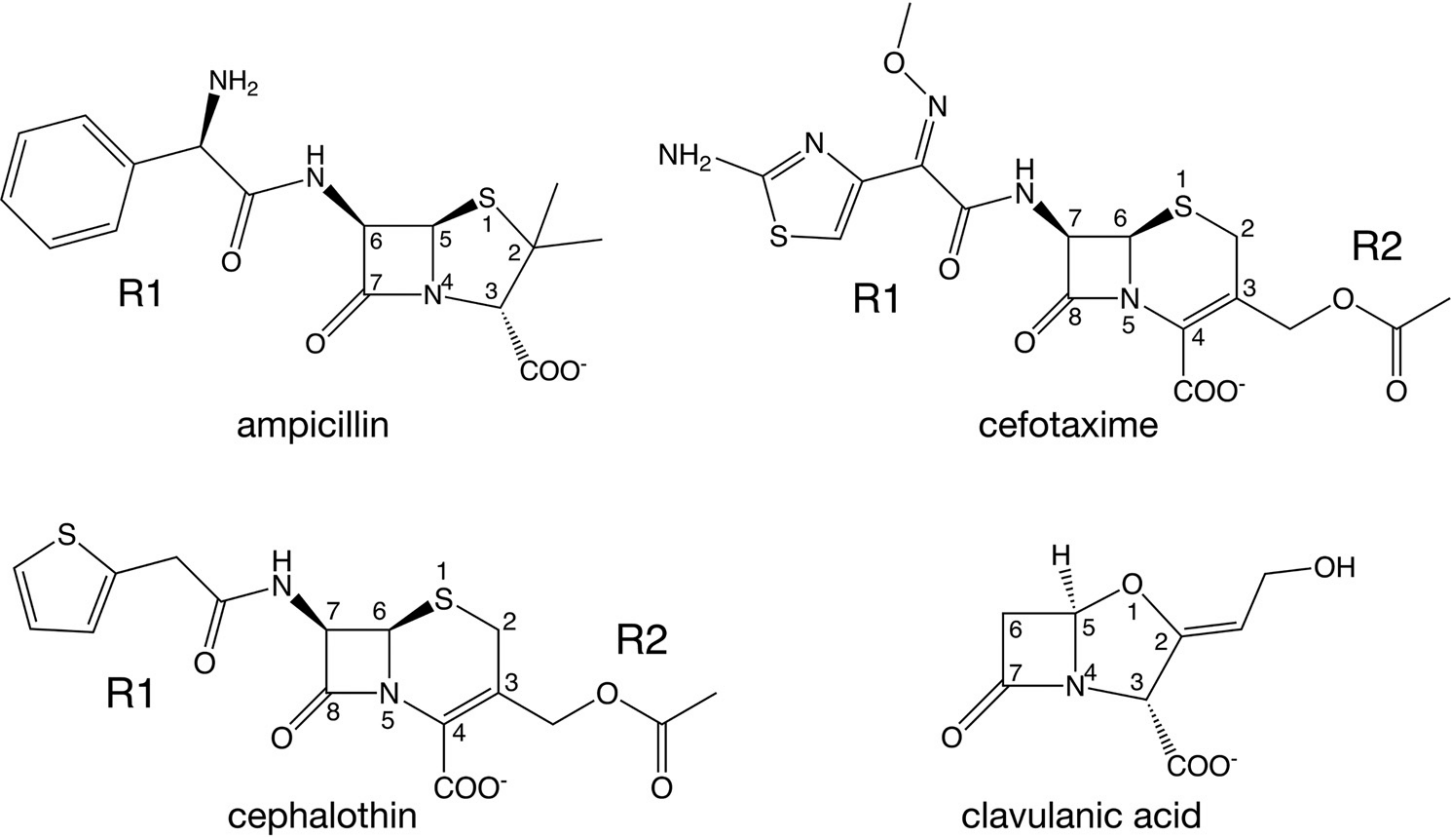
**β-Lactam antibiotics and inhibitor used in this study.** The R1 and R2 groups represent chemical substituents that vary within the class. Key atom positions are *numbered*.

There is also evidence that Lys^234^ has a role in determining the substrate specificity and inhibitor susceptibility of class A β-lactamases. Although lysine is conserved at position 234 among most class A enzymes, this residue is an arginine in a subclass of enzymes named carbenicillinases that includes the *Pseudomonas aeruginosa* PSE-4 β-lactamase (25). These enzymes are characterized by the ability to efficiently hydrolyze carbenicillin, and thus, they exhibit an altered substrate specificity compared with the canonical TEM-1 β-lactamase (25, 26). In addition, the natural variants SHV-72 and SHV-84 contain the K234R substitution, and these enzymes display reduced susceptibility to the β-lactamase inhibitor clavulanic acid and lower catalytic efficiency for cephalosporin hydrolysis (15, 27). Finally, the CTX-M-106 and -107 natural variants contain the K234R substitution, and selection of CTX-M-14 mutants for increased resistance to clavulanic acid led to the isolation of a K234R mutant (19, 28). Subsequent characterization of the enzyme revealed decreased susceptibility to clavulanic acid and decreased hydrolysis of cephalosporins (28).

To gain a mechanistic understanding of how Lys^234^ and its various substitutions differentially alter CTX-M β-lactamase substrate specificity and the associated resistance profile, we examined the sequence requirements at position 234 for penicillin and cephalosporin hydrolysis using codon randomization mutagenesis, which revealed that lysine is required for cephalosporin hydrolysis, whereas either lysine or arginine is consistent with penicillin hydrolysis. Subsequent purification of the K234R enzyme and kinetic analysis showed that the catalytic efficiency for ampicillin hydrolysis was unchanged, whereas *k*_cat_/*K_m_* was 100-fold lower for cefotaxime hydrolysis. In addition, kinetics experiments revealed a 1,500-fold reduced acylation rate for the K234R enzyme, consistent with disruption of the Ser^130^-mediated protonation of the β-lactam leaving group nitrogen. Further, X-ray structures of the K234R apoenzyme and complexes with ampicillin and cefotaxime revealed an altered conformation of Ser^130^, again consistent with a defect in acylation. Finally, we show that clavulanic acid is a less potent inhibitor of the K234R enzyme and that the change in conformation of Ser^130^ is correlated with the loss of inhibitor potency. These findings suggest that clavulanic acid should be paired with an oxyimino-cephalosporin rather than a penicillin to slow the evolution of resistance because mutations that alter the presence or position of Ser^130^ block cephalosporin hydrolysis.

## Results

### Amino acid sequence requirements at residue 234 differ for penicillins versus cephalosporins

**Figure 4:**
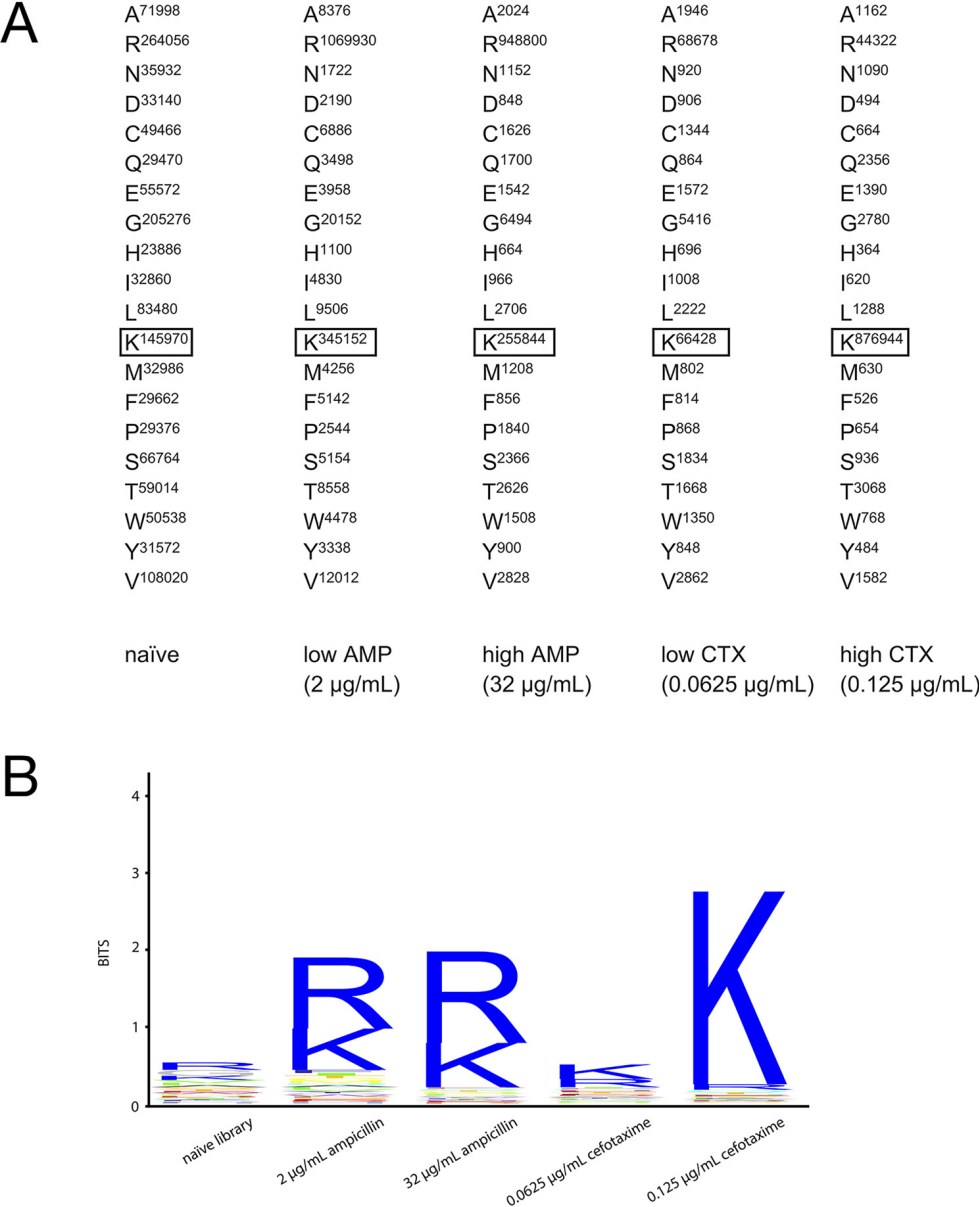
**NGS results for K234X library clones before and after selection for ampicillin or cefotaxime resistance.***A*, NGS sequencing read counts from the K234X naive library and after low- and high-concentration selections in media containing ampicillin or cefotaxime. The number of occurrences of reads encoding each amino acid are shown. The WT amino acid that occurred in the sequenced library is *boxed* in each experiment. *AMP*, ampicillin; *CTX*, cefotaxime. *B*, sequence logos derived from the read count data shown in *A*. The *height* of the *letter* is proportional to the occurrences of each amino acid type. The results for the naive library and each antibiotic selection are indicated *below* each experiment.

To examine the role of Lys^234^ in CTX-M enzyme catalysis and substrate specificity, we determined the amino acid sequence requirements at position 234 for penicillin and cephalosporin hydrolysis. We reasoned that, if the sequence requirements are similar for each substrate, the position is not a determinant of substrate specificity (*i.e.* substitutions do not differentially affect penicillin *versus* cephalosporin hydrolysis) (29). For this purpose, we used site-directed mutagenesis to randomize the DNA sequence of the codon to create a library of all possible substitutions at the position (30). The library was introduced into *Escherichia coli*, and the resultant colonies were pooled and grown in media containing high concentrations of ampicillin or cefotaxime that selected for high enzyme activity as well as low concentrations where only partial activity was required for bacteria to survive the selection (Figure 3, Figure 4). Plasmid DNA was recovered from each culture after overnight growth under selective conditions, and the region of the *bla* gene containing the residue 234 region was amplified by PCR with a 7-bp barcode at the 5′-end of each primer to uniquely identify each experiment. The PCR products were pooled, and next-generation sequencing (NGS) was performed to determine the spectrum of amino acids that are consistent with CTX-M-14 function under each selective condition.

The deep-sequencing results revealed that the naive library without antibiotic selection was diverse and all amino acids were present at similar frequencies (Fig. 4*A*). In contrast, arginine and lysine were significantly enriched after selection in both high- and low-ampicillin conditions, whereas all other amino acids were present at low frequency (Fig. 4*B*). The deep-sequencing results for the low-concentration cefotaxime selection showed that lysine and arginine were enriched but not to the extent observed for the ampicillin selections, as other amino acids were present at significant frequencies (Fig. 4*A*). At high cefotaxime concentrations, however, lysine was dominant and present at a much higher frequency than other amino acids, including arginine (Fig. 4*B*). Taken together, these results suggest that either lysine or arginine at position 234 supports high levels of ampicillin resistance and, presumably, high catalytic efficiency for ampicillin hydrolysis. In contrast, only lysine appears to provide high-level resistance and high levels of enzyme activity toward cefotaxime. Therefore, the identity of the amino acid at position 234 strongly influences the penicillinase *versus* cephalosporinase characteristics of the CTX-M enzyme.

### CTX-M K234R is a poor cephalosporinase due to slow substrate turnover

The dominance of lysine in random library selections and subsequent deep-sequencing results at high cefotaxime concentrations predicts that the CTX-M-14 K234R-substituted enzyme should display high catalytic efficiency for ampicillin hydrolysis but reduced activity toward cefotaxime. To test this hypothesis, we purified the K234R enzyme and performed steady-state kinetic analysis with ampicillin and cefotaxime as substrates (Fig. 3 and Table 1). The *k*_cat_ for ampicillin hydrolysis for the K234R enzyme is increased 5-fold and *K_m_* is increased 9-fold, whereas *k*_cat_/*K_m_* is only modestly changed compared with the WT enzyme (Table 1). With cefotaxime as substrate, *k*_cat_ for the K234R enzyme is drastically reduced (500-fold), whereas *K_m_* is reduced 6-fold and *k*_cat_/*K_m_* is reduced 80-fold compared with the WT enzyme (Table 1). The kinetic results are consistent with the observation of both lysine and arginine at this position when the selection is for ampicillin resistance but the occurrence of only WT lysine with selection for cefotaxime resistance.

**Table 1:** Steady-state kinetic parameters for the WT CTX-M-14 and K234R enzymes

Enzyme	Ampicillin			Cefotaxime			Clav acid^a^ K_i_
	*k*_cat_	*K_m_*	*k*_cat_/*K_m_*	*k*_cat_	*K_m_*	*k*_cat_/*K_m_*	
	*s*^−*1*^	μ*m*	*m*^−*1*^*s*^−*1*^	*s*^−*1*^	μ*m*	*m*^−*1*^*s*^−*1*^	μ*m*
WT CTX-M-14	67 ± 3	120 ± 10	5.6 × 10^5^ ± 2 × 10^4^	100 ± 1	66 ± 3	1.5 × 10^6^ ± 1 × 10^5^	0.08 ± 0.001
K234R	330 ± 33	1,050 ± 170	3.1 × 10^5^ ± 3 × 10^4^	0.2 ± 0.01	11 ± 4	1.8 × 10^4^ ± 5,000	0.39 ± 0.02
S130A	30 ± 2	30,700 ± 2,440	1.0 × 10^3^ ± 2 × 10^2^	0.1 ± 0.003	260 ± 13	3.8 × 10^2^ ± 10	31 ± 7
S130A/K234R	ND^b^	ND	1.1 × 10^3^ ± 20	0.0005 ± 0.0001	120 ± 17	4.2 ± 0.3	270 ± 20


a Clavulanic acid *K_i_* measured with ampicillin as indicator substrate.

b ND, not determined.

In the β-lactamase kinetic scheme, the observed *k*_cat_ reflects the magnitude and relationship between the acylation (*k*_2_) and deacylation (*k*_3_) rates. Therefore, the greatly reduced *k*_cat_ for cefotaxime hydrolysis is the result of a decrease in *k*_2_ and/or *k*_3_ (10, 31) (Fig. 2*A*). *k*_cat_/*K_m_* reflects the rates of reactions up to and including the first irreversible step (32), and for serine β-lactamases it therefore reports on the steps up to the formation of the acyl-enzyme intermediate (10) (Fig. 2*A*). Thus, the 80-fold decrease for K234R could be due to a decreased acylation rate and/or decreased affinity for cefotaxime. However, the reduction of *k*_cat_ by 500-fold suggests that the major effect of the K234R mutation is to decrease the rate of acylation of cefotaxime.

We were next interested in determining the effect of the K234R substitution on the hydrolysis of cephalosporins other than cefotaxime. We examined kinetic parameters for several oxyimino-cephalosporins that have an identical R1 group but differ from the acetyloxymethyl R2 group found at the C3 position of cefotaxime (Table S1). Cefpodoxime has a methoxymethyl R2 group at C3 and is hydrolyzed by the WT CTX-M-14 enzyme with kinetic parameters very similar to those observed for cefotaxime (Table S1). The kinetic parameters for hydrolysis of cefpodoxime by the K234R enzyme were also found to be very similar to those observed for cefotaxime, with a 430-fold decrease in *k*_cat_, a 9-fold decrease in *K_m_*, and a 50-fold decrease in *k*_cat_/*K_m_*. Therefore, the difference in R2 group between cefotaxime and cefpodoxime had no effect on catalysis. Ceftriaxone also has the same R1 group as cefotaxime but contains a methyl-dioxo-triazine ring linked to C3 by a sulfanylmethyl group. Despite the different R2 group, hydrolysis of ceftriaxone by the WT enzyme exhibits similar kinetic parameters as cefotaxime (Table S1). In addition, the effect of the K234R substitution is similar to that observed for cefotaxime, with a 400-fold decrease in *k*_cat_, a 2-fold decrease in *K_m_*, and a 170-fold decrease in *k*_cat_/*K_m_*. This is consistent with the results for cefpodoxime and again shows that the R2 group does not alter the impact of the K234R substitution. Further, we examined the kinetic parameters for hydrolysis of ceftizoxime, which does not have an R2 group and contains only a hydrogen at C3. In contrast to the other substrates, the kinetic parameters for ceftizoxime hydrolysis by the WT enzyme are different from those observed for cefotaxime, with a 2-fold higher *k*_cat_, a 45-fold higher *K_m_*, and a 20-fold lower *k*_cat_/*K_m_* value (Table S1). Despite these differences, the impact of the K234R substitution is similar to that observed for cefotaxime, with a 320-fold decrease in *k*_cat_, a 8-fold decrease in *K_m_*, and a 40-fold decrease in *k*_cat_/*K_m_* compared with WT CTX-M-14 (Table S1). In aggregate, the enzyme kinetics results for the various oxyimino-cephalosporins with identical R1 groups but different R2 groups suggest that the effect of the K234R substitution is not mediated through the R2 group.

We also examined the influence of the K234R substitution on hydrolysis of nonoxyimino-cephalosporins. Cephalothin is a second-generation cephalosporin with an R1 group that differs from cefotaxime but contains an identical R2 group at the C3 position (Fig. 3). Cephalothin is rapidly hydrolyzed by WT CTX-M-14, with a *k*_cat_ value nearly 7-fold higher and *k*_cat_/*K_m_* value ∼3-fold higher than that observed for cefotaxime (Table S1). The trend of the effects on kinetic parameters for cephalothin hydrolysis by the K234R enzyme is similar to that observed for cefotaxime, with a 100-fold decrease in *k*_cat_, a 20-fold decrease in *K_m_*, and a 6-fold decrease in *k*_cat_/*K_m_*_,_ suggesting that the effect of the K234R substitution is also not mediated through the R1 group. Taken together, these results suggest that the substitution is not acting through the R1 or R2 group but rather through a conserved feature of cephalosporins, namely the configuration of the β-lactam amide nitrogen relative to the C4 carboxylate group compared with that in penicillins.

### Single-turnover kinetics reveals that K234R slowly acylates cefotaxime

To further understand how the K234R substitution hinders cefotaxime hydrolysis, we determined the rate constants for acylation and deacylation. Steady-state kinetics experiments cannot estimate individual rate constants. Therefore, we performed single-turnover kinetics with an excess of CTX-M-14 enzyme over cefotaxime substrate to determine the acylation rate (*k*_2_) (33, 34, 35). We then calculated the deacylation rate (*k*_3_) using the experimental values of *k*_2_ and *k*_cat_ (Fig. 2*A*). Although the CTX-M enzymes have been extensively studied, the acylation and deacylation rates have not yet been determined for any β-lactam substrate. Consequentially, the rate-limiting step for the WT enzyme CTX-M-14 enzyme remains unknown. Therefore, we first determined the acylation rate for the CTX-M-14 enzyme and subsequently determined the acylation rate for the K234R mutant enzyme.

**Figure 5:**
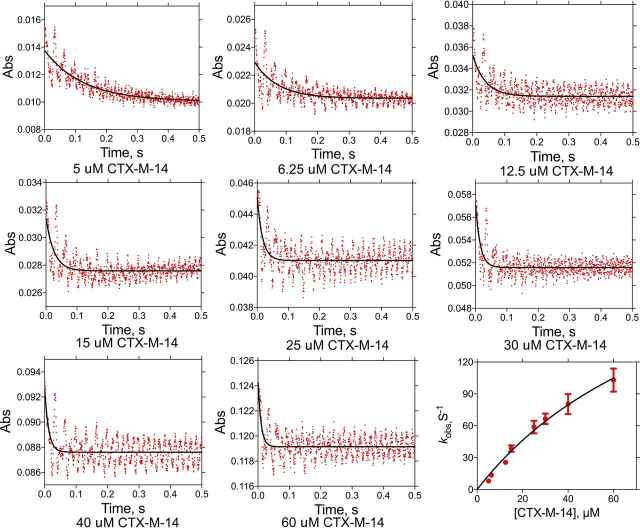
**Single-turnover kinetic analysis of WT CTX-M-14 hydrolysis of cefotaxime.** 5 μm cefotaxime was used with increasing concentrations of CTX-M-14 enzyme as indicated *below* each plot. Absorbance is shown on the *y* axis and time in seconds on the *x* axis. At the *bottom right* is the fit of the *k*_obs_ values *versus* the CTX-M-14 enzyme concentrations to a hyperbola to obtain the acylation rate (*k*_2_).

Single-turnover kinetics results with the CTX-M-14 enzyme revealed an acylation rate (*k*_2_) of 210 s^−1^ (Table 2 and Fig. 5). Using the equation for *k*_cat_ based on the kinetic scheme, the deacylation rate (*k*_3_) was calculated to be 190 s^−1^ (Fig. 2*A*). Therefore, the acylation and deacylation rates are similar, and there is not a strongly rate-limiting step for hydrolysis of cefotaxime by the WT enzyme. In addition, the fit of *k*_obs_ values indicates that the dissociation constant (*K_s_*) for cefotaxime is 74 μm (Fig. 5). Based on the kinetic scheme, *K_m_* approximates *K_s_* when acylation (*k*_2_) is rate-limiting. In this case, neither acylation or deacylation (*k*_3_) is rate-limiting, and the *K_m_* value based on steady-state kinetics, 66 μm, is relatively close to the value of *K_s_*.

**Table 2:** Acylation and deacylation rates for cefotaxime hydrolysis

	*k*_2_^a^	*k*_3_^a^	*K_D_*^a^
	*s*^−*1*^	*s*^−*1*^	μ*m*
CTX-M-14	210 ± 12	190 ± 35^b^	74 ± 12
K234R	0.14 ± 0.01	ND^c^	42 ± 8


a *k*_2_ and *K_D_* were determined based on fitting single-turnover results (*k*_obs_) to the equation, *k*_obs_ = (*k*_2_ × *E*)/(*K_D_* + *E*). *k*_3_ was calculated from *k*_2_ and *k*_cat_ using the equation, *k*_cat_ = (*k*_2_ × *k*_3_)/(*k*_2_ + *k*_3_).

b The error on *k*_3_ was propagated through the equation, *k*_3_ = (*k*_2_ × *k*_cat_)/(*k*_cat_ − *k*_2_), by calculating the appropriate error for each term (see “Experimental procedures”).

c Not determined.

**Figure 6:**
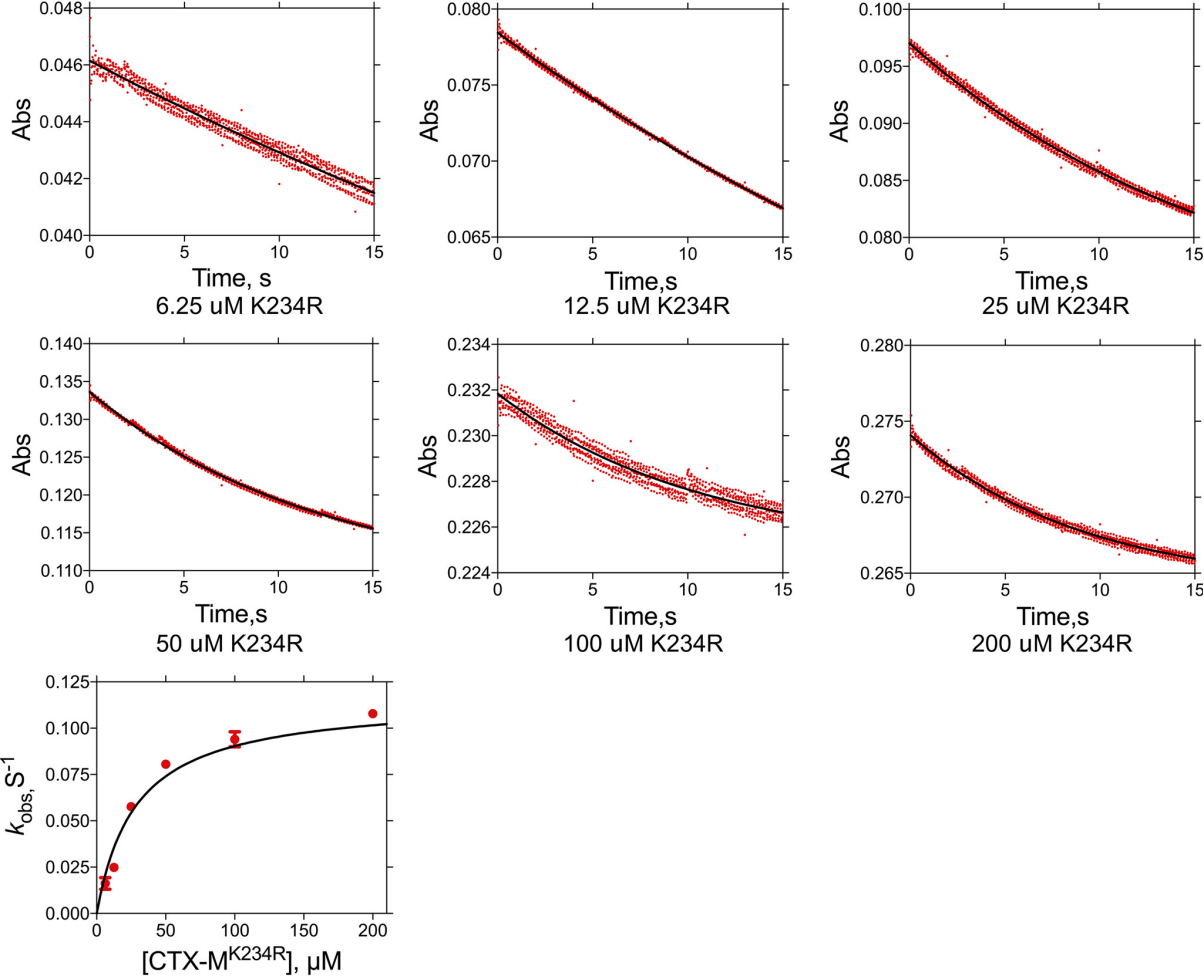
**Single-turnover kinetic analysis of CTX-M-14 K234R hydrolysis of cefotaxime.** 2.5 μm cefotaxime was used with increasing concentrations of the K234R enzyme as indicated *below* each plot. Details are as described for Fig. 5.

The acylation rate (*k*_2_) for the K234R mutant enzyme with cefotaxime as substrate was determined by single-turnover kinetics experiments to be 0.14 s^−1^ (Table 2 and Fig. 6). Therefore, the K234R substitution results in a 1,500-fold decrease in the rate constant for acylation. It is not possible to estimate the deacylation rate, but, based on the *k*_cat_ value of 0.2 s^−1^, it must be ≥0.2 s^−1^ (Table 1). Single-turnover kinetics experiments were not performed for the K234R enzyme with ampicillin because the *k*_cat_ value of 330 s^−1^ indicates that the reaction is too fast to be measured accurately. However, based on the *k*_cat_ value, *k*_2_ and *k*_3_ must be ≥330 s^−1^. Therefore, the K234R substitution reduces the acylation rate for cefotaxime by 1,500-fold but increases the acylation rate for ampicillin by at least 5-fold. The net effect is a change in the substrate profile that favors ampicillin over cefotaxime hydrolysis by the K234R enzyme, due largely to a very slow acylation reaction for cefotaxime (Table 1, Table 2).

The single-turnover results indicate that the acylation step is very slow for the K234R enzyme with cefotaxime as substrate but not with ampicillin. Residue 234 is adjacent to Ser^130^, which is thought to protonate the leaving group nitrogen of the amide bond during the acylation reaction (8, 9) (Fig. 1). The β-lactam ring is fused to a five-membered ring for penicillins and a six-membered ring for cephalosporins, which changes the distance between the C3/C4 carboxylate and the carbonyl oxygen of the amide bond that, in turn, alters positioning of the leaving group nitrogen relative to Ser^130^ in penicillins *versus* cephalosporins (Fig. 2, *B* and *C*). Therefore, a hypothesis to explain the different rates of acylation of penicillins *versus* cephalosporins is altered structural positioning of the substrates relative to Ser^130^, resulting in more effective acylation for penicillin.

### CTX-M K234R exhibits an altered conformation of the Ser^130^ proton shuttle

To further investigate the molecular basis of the slow acylation of cefotaxime due to the K234R substitution, we determined the X-ray crystal structures of the CTX-M-14 E166A/K234R apoenzyme as well as the E166A/K234R acyl-enzyme complexes with ampicillin and cefotaxime.

**Figure 7:**
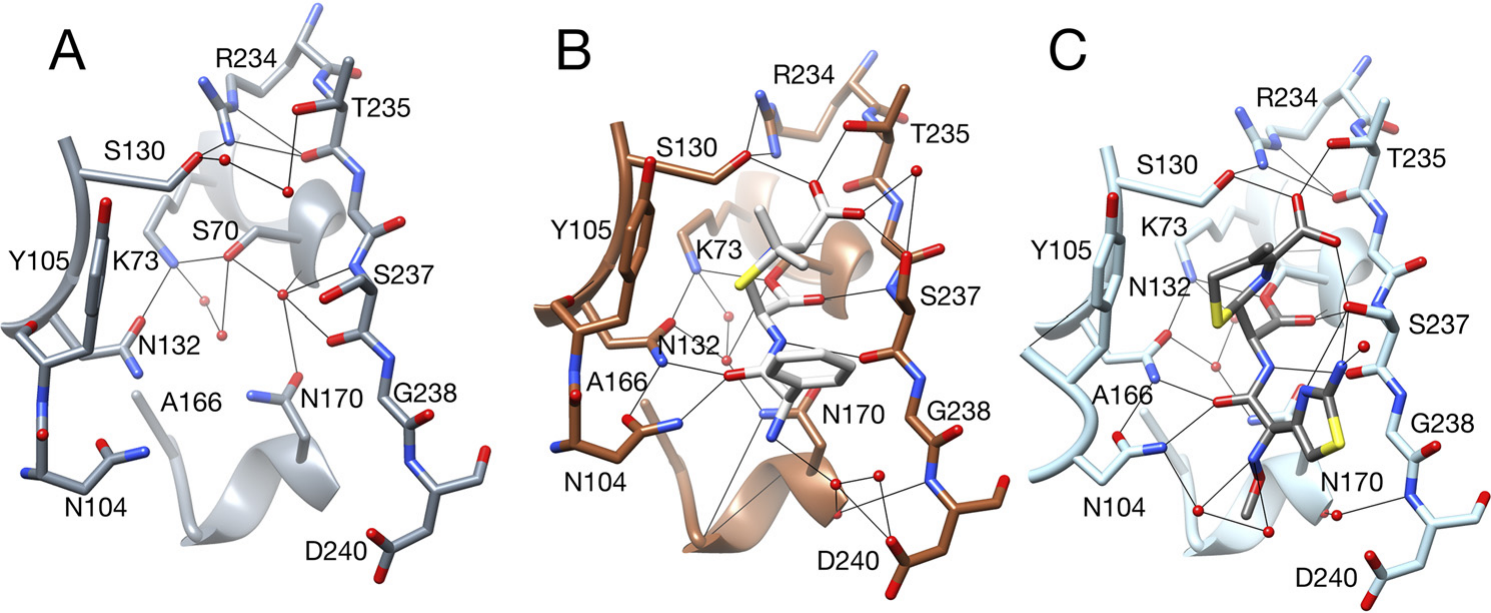
**X-ray crystal structures of the CTX-M-14 E166A/K234R apoenzyme and acyl-enzyme complexes with ampicillin and cefotaxime.***A*, active-site region of the E166A/K234R apoenzyme. Note that the Ser^130^ side chain is in an alternate conformation and is predicted to hydrogen-bond with the guanidinium group of Arg^234^ and a water molecule. The catalytic water molecule is present and hydrogen-bonded to Ser^70^, and a water molecule occupies the oxyanion hole. Hydrogen bonds are shown as *thin black lines*. Carbon atoms are shown in *gray*, nitrogen in *blue*, and oxygen in *red*. *B*, active-site region of the E166A/K234R enzyme with Ser^70^ forming a covalent acyl-enzyme with ampicillin. Ser^130^ forms two hydrogen bonds with Arg^234^ as well as the ampicillin C3 carboxylate oxygen. The catalytic water is present and is predicted to hydrogen-bond to Asn^132^ and Asn^170^. Carbon atoms are shown in *brown*. *C*, active-site region of the E166A/K234R enzyme with Ser^70^ forming a covalent acyl-enzyme with cefotaxime. Ser^130^ forms two hydrogen bonds with Arg^234^ as well as the cefotaxime C4 carboxylate oxygen. As with the E166A/K234R/AMP structure, the catalytic water is present and predicted to hydrogen-bond to Asn^132^ and Asn^170^. Carbon atoms are shown in *light blue*.

The structure of the E166A/K234R apoenzyme was determined to 1.8 Å resolution and contained eight molecules in the asymmetric unit (Table S2). In each of the eight chains, the position of the side chain and associated guanidium group of Arg^234^ is similar and is oriented in the same manner as is Lys^234^ in the structure of WT CTX-M-14 (1YLT) (Figs. 7*A* and Fig. S1). One nitrogen of the guanidinium group occupies the same position as the Lys^234^ Nζ atom. A clear difference in the structures is that the hydroxyl group of Ser^130^ is shifted in position relative to that in WT and is directed toward the guanidinium group of Arg^234^ in all eight chains (Figs. 7*A* and Fig. S1). This rotamer has an χ_1_ value of −60° compared with −150° found in WT CTX-M-14 and nearly all class A β-lactamases. Interestingly, this serine rotamer is preferred in general surveys of protein structures. In this position, the serine hydroxyl can form hydrogen bonds to a water molecule and the guanidinium nitrogen of Arg^234^ (Fig. 7*A*).

**Figure 8:**
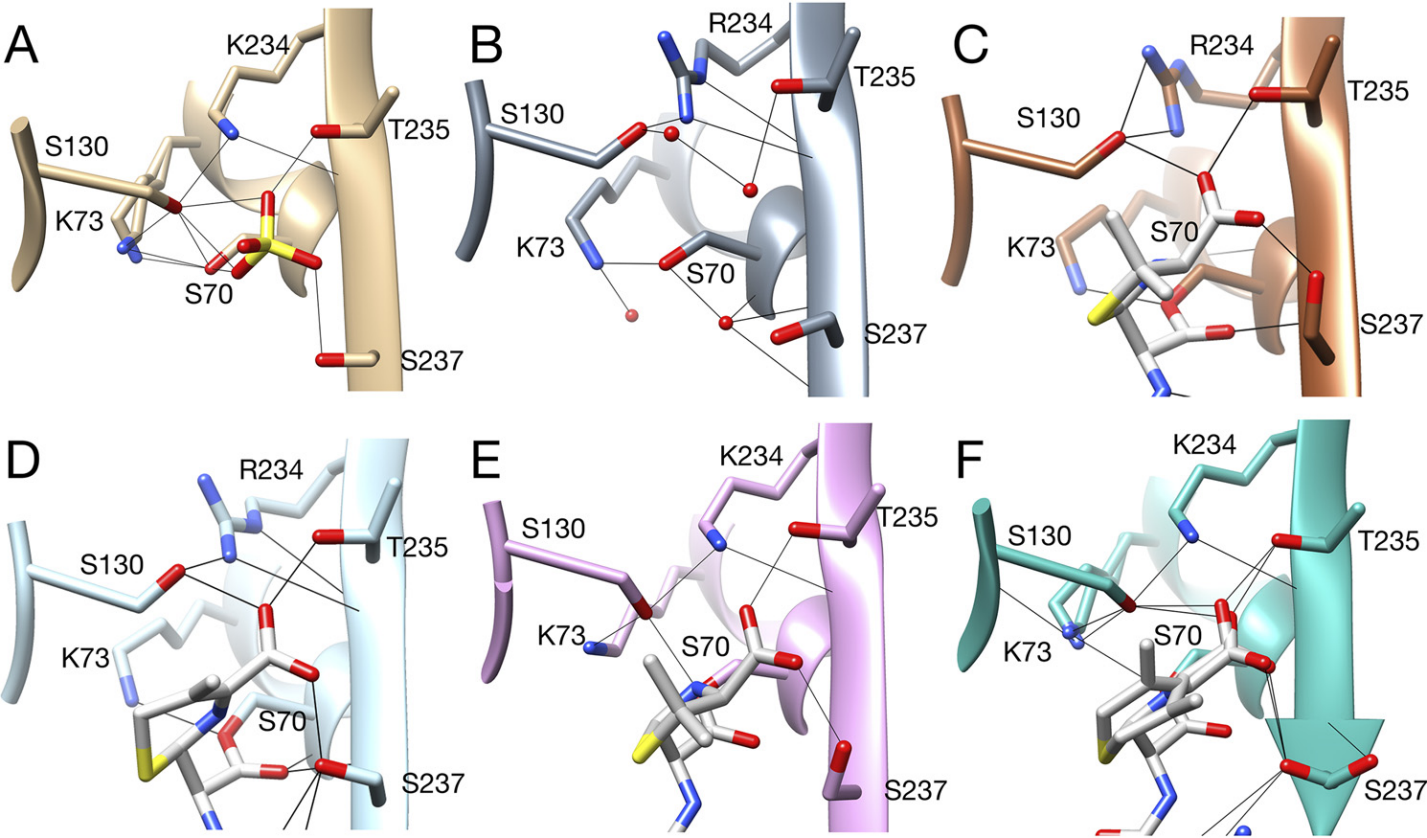
**CTX-M-14 active-site structures focused on the Lys^234^ region.***A*, WT CTX-M-14 active site (PDB entry 1YLT) showing key residues interacting with Lys^234^. The Ser^130^ side chain is in the χ_1_ −141° rotamer conformation and forms hydrogen bonds with Lys^73^, Lys^234^, Ser^70^, and a sulfate molecule. *B*, E166A/K234R apoenzyme structure. The Ser^130^ side chain is in the χ_1_ −60° rotamer conformation and forms hydrogen bonds with Arg^234^ and a water but not Lys^73^. *C*, E166A/K234R/AMP structure showing that the Ser^130^ side chain is in the χ_1_ −60° rotamer conformation and forms two hydrogen bonds with Arg^234^ and a hydrogen bond with the C3 carboxylate of ampicillin but not with Lys^73^. *D*, E166A/K234R/CTX structure showing that the Ser^130^ side chain is in the χ_1_ −60° rotamer conformation and forms hydrogen bonds with Arg^234^ and the cefotaxime C4 carboxylate. *E*, CTX-M Toho-1 E166A acyl-enzyme structure with benzylpenicillin. Ser^130^ is in the χ_1_ −140° rotamer conformation and forms hydrogen bonds with Lys^73^, Lys^234^, and the N4 nitrogen of benzylpenicillin. *F*, CTX-M Toho-1 E166A acyl-enzyme structure with cefotaxime. Ser^130^ is in the χ_1_ −140° rotamer conformation and is predicted to form hydrogen bonds with Lys^73^, Lys^234^, and the N5 nitrogen of cefotaxime.

The structure of the E166A/K234R enzyme acylated by ampicillin was determined to 1.6 Å resolution with one molecule in the asymmetric unit (Table S2). The E166A substitution blocks the deacylation reaction, which allows trapping of the acyl-enzyme intermediate (6, 36). As anticipated, the structure reveals ampicillin covalently bound to the enzyme via Ser^70^, as indicated by an *F_o_* − *F_c_* omit map (Fig. S2A). The carbonyl oxygen of the acyl-group makes hydrogen bonds to Ser^70^ and Ser^237^ main-chain nitrogens to form the oxyanion hole (Fig. 7*B*). The C3 carboxyl group of ampicillin also forms hydrogen bonds to the side chains of Ser^130^, Thr^235^, and Ser^237^. The Ser^130^ OH is likely a hydrogen bond donor to the C3 carboxylate oxygen with a distance of 2.8 Å (Fig. 7*B*). In addition, the Ser^130^ hydroxyl group is oriented toward Arg^234^ and forms two hydrogen bonds with guanidinium nitrogens (Figs. 7*B* and 8). This Ser^130^ rotamer is the same as that observed in the E166A/K234R apoenzyme with a χ_1_ value of −60° (Fig. 8, *B* and *C*). The Ser^130^ Oγ is 3.6 Å from the leaving group N4 nitrogen of the β-lactam ring and at an unfavorable angle for hydrogen bonding. The E166A/K234R/AMP structure is superimposable with the structure of the CTX-M Toho-1 E166A β-lactamase acyl-enzyme complex with benzylpenicillin (1IYQ) with the major differences being the primary amine on the R1 group of ampicillin that is not present in benzylpenicillin and the rotamer conformation of Ser^130^ (37, 38) (Fig. 8, *C* and *E*). The ampicillin side chain amine is located at the bottom of the active site above Asn^170^ and forms a hydrogen bond with a water molecule (Fig. 7*B*). Ser^130^ in the Toho-1 structure is in the commonly observed χ_1_ −150° rotamer conformation with the Oγ forming a hydrogen bond with the benzylpenicillin leaving group N4 nitrogen at a distance of 2.9 Å (Fig. 8*E*). A structure of intact benzylpenicillin substrate in complex with a CTX-M-9 S70G enzyme (3HUO) also shows the Ser^130^ Oγ forming hydrogen bonds with the Lys^234^ Nζ at 3 Å and with the β-lactam leaving group nitrogen within weak hydrogen-bonding distance of 3.4 Å (Fig. S3A). Therefore, in available CTX-M enzyme structures in complex with penicillins, the Ser^130^ hydroxyl group is directed toward the leaving group N4 nitrogen except for the E166A/K234R/AMP structure, where it is directed away from the nitrogen and toward Arg^234^. Further, in the proposed β-lactamase mechanism, a proton is shuttled from Lys^73^ Nζ to Ser^130^ Oγ and subsequently to the leaving group N4 nitrogen (9) (Fig. 1). In the −64° χ_1_ rotamer conformation of Ser^130^ in the E166A/K234R/AMP structure, the serine oxygen is 5.7 Å from the Lys^73^ Nζ, which is too distant for proton transfer (Fig. 7*B*). In contrast, in the Toho-1 E166A/benzylpenicillin structure, the Ser^130^ Oγ is 2.6 Å from Lys^73^ and forms a hydrogen bond with Lys^73^ Nζ (Fig. 8*E*) (37, 38). Taken together, the structures suggest that the Ser^130^ side chain in the K234R mutant would need to flip back to the −150° χ_1_ rotamer conformation during the catalytic cycle to allow protonation of the ampicillin leaving group N4 nitrogen.

The structure of the E166A/K234R enzyme acylated by cefotaxime was determined at 1.4 Å resolution (Fig. S2B and Table S2). As with the ampicillin structure, the carbonyl oxygen of the β-lactam in the cefotaxime acyl-enzyme is in position to hydrogen-bond with the main-chain Ser^70^-N and Thr^237^-N in the oxyanion hole (Fig. 7*C*). The C3-carboxylate group is positioned for hydrogen bonds to Ser^130^, Thr^235^, and Ser^237^, similar to the ampicillin-acyl-enzyme. Also, similar to the E166A/K234R/AMP structure, Ser^130^ is in a −49° χ_1_ rotamer conformation, and the hydroxyl group forms two hydrogen bonds with the Arg^234^ guanidinium (Fig. 7*C*). The Ser^130^ Oγ is 3.9 Å from the cefotaxime N5 leaving group nitrogen, which is outside of the hydrogen-bonding range. In addition, the Ser^130^ Oγ is 7.8 Å from the Lys^73^ Nζ and is not in position to accept a proton as proposed in the catalytic mechanism (8, 9) (Fig. 7*C*). In contrast, in the structure of the Toho-1 E166A cefotaxime acyl-enzyme complex (1IYO), Ser^130^ is in a −145° χ_1_ rotamer conformation and forms a 2.8-Å hydrogen bond with Lys^234^ Nζ (37). In this structure, Lys^73^ is in two conformations, and the Ser^130^ Oγ is 2.7 and 3.1 Å from the Lys^73^ Nξ for these conformations (Fig. 8*F*). The conformations of Lys^73^ have been proposed to be integral to the function of Lys^73^ in shuttling protons to Ser^130^, and the conformation at 2.7 Å from Ser^130^ has been suggested to be active in proton transfer (8, 9, 23). In addition, the Ser^130^ Oγ in the Toho-1 E166A/CTX structure is 3.0 Å from the β-lactam leaving group N5 nitrogen, which is within the hydrogen-bonding range.

### Ser^130^ contributes to cefotaxime but not ampicillin turnover

The structural results described above suggest the Ser^130^ residue occupies an alternate rotamer conformation in the presence of the K234R substitution. This is the case in the apoenzyme as well as the acyl-enzyme structures for ampicillin and cefotaxime. This does not offer a ready explanation for an increased rate of acylation for ampicillin and a large decrease in the cefotaxime acylation rate. To investigate this puzzling finding further, we examined the effect of removing the hydroxyl group from Ser^130^ via an S130A substitution made in the context of the WT enzyme and in the presence of the K234R substitution.

The S130A substitution alone displays remarkably different effects on kinetic parameters for ampicillin *versus* cefotaxime hydrolysis. For ampicillin hydrolysis, *k*_cat_ is reduced only 2-fold compared with the WT enzyme, whereas for cefotaxime, *k*_cat_ is reduced 1,000-fold (Table 1). Because *k*_cat_ places a lower limit on *k*_2_ and *k*_3_, this result indicates that the removal of the Ser^130^ hydroxyl group, which eliminates the ability of the residue to transfer a proton to the β-lactam leaving group nitrogen, has only modest effects on the ampicillin acylation rate. In contrast, removal of the Ser^130^ hydroxyl drastically reduces *k*_2_ and/or *k*_3_ for cefotaxime hydrolysis. Clearly, the catalysis of ampicillin hydrolysis (acylation/deacylation) does not require the hydroxyl group of Ser^30^ as a proton shuttle, and therefore the altered conformation of Ser^130^ in the K234R mutant does not have any effect. In contrast, Ser^130^ is critical for cefotaxime acylation, and consequently the change in conformation with the hydroxyl turned away from Lys^73^ and the β-lactam N5 nitrogen drastically reduces catalysis (Table 1, Table 2).

Another striking difference in the effect of the S130A substitution is that it results in a 260-fold increase in *K_m_*, to 30 mm, for ampicillin hydrolysis but only a 4-fold increase in *K_m_*, to 260 μm, for cefotaxime hydrolysis (Table 1). *K_m_* is not necessarily equal to the *K_D_* for substrate binding but does reflect the balance between the rate of turnover and the rate of substrate binding. The result suggests that the Ser^130^ hydroxyl group makes an important contribution to ampicillin binding but much less so for cefotaxime. Thus, the Ser^130^ hydroxyl group contributes strongly to ampicillin binding but not turnover and contributes strongly to cefotaxime turnover but not binding.

We next examined the kinetic parameters for ampicillin and cefotaxime hydrolysis by the S130A/K234R double mutant enzyme. The K234R substitution increases *k_c_*_at_ for ampicillin hydrolysis by 5-fold, and the S130A substitution reduces *k*_cat_ by a modest 2-fold (Table 1). The *K_m_* for the double mutant for ampicillin was too high to measure (>30 mm), but *k*_cat_/*K_m_* was the same as the S130A single mutant, indicating that *k*_cat_ is ≥30 s^−1^. Therefore, the S130A substitution does not substantially change *k*_cat_ when added to the K234R mutant, which is consistent with Ser^130^ not being required for acylation of ampicillin (Table 1). Both the K234R and S130A substitutions result in large increases in *K_m_* and reductions in *k*_cat_/*K_m_*, suggesting that Ser^130^ contributes to ampicillin binding and that the K234R substitution disrupts binding (Table 1). The finding that the double mutant has a *K_m_* value that is too high to measure provides further evidence supporting these roles.

The K234R substitution reduced *k*_cat_ for cefotaxime hydrolysis 500-fold compared with the WT enzyme via a large decrease in the acylation rate (Table 1, Table 2). The addition of S130A to K234R to create the double mutant results in a further 400-fold reduction in *k*_cat_ compared with the K234R enzyme (Table 1). This shows that Ser^130^ contributes to the turnover of cefotaxime by the K234R enzyme, presumably by enabling the proton shuttle to the β-lactam N5 nitrogen, albeit at reduced efficiency compared with the WT enzyme. The double mutant also exhibits a 200-fold reduced *k*_cat_ value compared with the S130A mutant (Table 1). Therefore, the K234R substitution decreases *k*_cat_ even when the proton shuttle is disabled, possibly by interfering with the unknown mechanism by which the N5 nitrogen of cefotaxime is protonated in the absence of Ser^130^.

### S130A, K234R, and S130A/K234R enzymes exhibit reduced clavulanic acid inhibition potency

Inhibition of class A β-lactamases by clavulanic acid has been studied extensively and involves formation of an acyl-enzyme followed by secondary ring opening that occurs due to the presence of an oxygen leaving group at the 1-position of the five-membered ring fused to the β-lactam (11) (Fig. 3). This creates a linear imine that can follow multiple chemical paths, including formation of a decarboxylated imine, a *trans*-enamine, a *cis*-enamine, and an aldehyde, all covalently linked to Ser^70^ (11, 14, 39, 40). In addition, the decarboxylated imine reacts with the hydroxyl of Ser^130^ to form a derivative that is covalently linked to both Ser^70^ and Ser^130^ (11, 39, 40). As noted above, the K234R substitution has been identified in SHV and CTX-M natural variants. For SHV, this mutation provides resistance to clavulanic acid by increasing the *K_i_* for inhibition (15). The K234R substitution was also identified in CTX-M-14 after directed evolution studies to identify inhibitor-resistant mutants where it increased the IC_50_ for clavulanic acid inhibition (41). Therefore, we determined the *K_i_* for clavulanic acid inhibition of WT CTX-M-14 and the K234R enzyme. The *K_i_* for the WT enzyme was 80 nm, whereas for K234R it increased to 390 nm, as expected based on previous studies (Table 1). This can be rationalized based on the changed conformation of Ser^130^ to the χ_1_ ∼−60° rotamer with the serine Oγ turned toward Arg^234^, which would interfere with the covalent bond formed between a clavulanic acid acyl-enzyme fragmentation product and Ser^130^ that cross-links the inhibitor between Ser^70^ and Ser^130^ (11). In addition, we show above that the ampicillin *K_m_* is increased in the K234R enzyme and greatly increased in the S130A enzyme (Table 1). Clavulanic acid contains a β-lactam ring and a five-membered fused ring, similar to penicillins (Fig. 3), and thus the conformational change of Ser^130^ may also decrease binding affinity for clavulanic acid.

The S130G substitution has been identified in TEM-1 and SHV-1 variants from clinical strains resistant to clavulanic acid (11). Clavulanic acid resistance has been attributed to an increased *K_i_* for inhibitor binding and can also be rationalized because an end-product of clavulanic acid inhibition is the cross-linked molecule between Ser^70^ and Ser^130^ (11, 12, 14). We show above that the change in conformation of Ser^130^ to the χ_1_ ∼−60° rotamer is associated with reduced clavulanic acid potency in K234R. The absence of the hydroxyl group in the S130A enzyme would also be expected to decrease potency. As expected, the *K_i_* for inhibition of the S130A enzyme was 30 μm, a factor of 400 increase in *K_i_* (Table 1). Finally, we tested the S130A/K234R double mutant enzyme for inhibition by clavulanic acid and found a further decrease in potency to a *K_i_* of 270 μm, 3,500-fold above WT. Therefore, the K234R substitution decreases clavulanic acid potency even in the absence of a hydroxyl group at position 130. This is consistent with the fact that the cross-link between Ser^70^ and Ser^130^ by clavulanic acid is not the only end product of inhibition and other clavulanic acid fragments covalently attached to Ser^70^ contribute to inhibition (11).

## Discussion

Here we show that for CTX-M-14 β-lactamase, lysine at position 234 is required for hydrolysis of cefotaxime and more generally for the hydrolysis of oxyimino-cephalosporins. In contrast, either lysine or arginine at position 234 provides for efficient hydrolysis of ampicillin. Lys^234^ is adjacent to and forms a hydrogen bond with Ser^130^ (6, 8, 23). The hydrogen bond as well as the positive charge of Lys^234^ would lower the p*K_a_* of Ser^130^ to facilitate transfer of a proton to the leaving group nitrogen of the β-lactam ring. The p*K_a_* of a serine hydroxyl group is ∼13 and would not easily transfer a proton unless its p*K_a_* was perturbed by surrounding residues. Ser^130^ is flanked by Lys^73^ and Lys^234^ that are both positioned to lower the p*K_a_* of the hydroxyl group. Whereas both Lys and Arg carry a charge of +1 at neutral pH, the positive charge on arginine is delocalized within the π-bonded system of the guanidium ion (42). In contrast, the charge on lysine is focused on the Nζ atom. The more delocalized charge raises the p*K_a_* of arginine and decreases the effect on the p*K_a_* of the Ser^130^ hydroxyl, thereby lowering the efficiency of proton transfer and decreasing the acylation rate. However, this effect does not impact ampicillin acylation, suggesting that the polarization of Ser^130^ does not make a large contribution to ampicillin hydrolysis.

The X-ray structures of the E166A/K234R apoenzyme as well as the acyl-enzyme structures of E166A/K234R with ampicillin and cefotaxime suggest that the K234R substitution acts through changing the conformation of Ser^130^ compared with WT CTX-M-14 or other enzymes, such as CTX-M-9, -15, -16, -27, and -96 and Toho-1 (Table 3). Serine largely occupies three rotamers in protein structures (43, 44). In the large majority of CTX-M enzymes whose structures are available, the Ser^130^ χ_1_ dihedral angle is ∼−150° (Table 3 and Fig. 8). In this position, the Ser^130^ hydroxyl group is positioned to exchange protons with both Lys^73^ and the N4 β-lactam nitrogen (9, 45). In the E166A/K234R apoenzyme and acyl-enzyme structures, however, Ser^130^ is in an alternate rotamer with χ_1_ ∼−60°, where it is not in position for the proton shuttle (Table 3). The altered conformation of Ser^130^, however, does not hinder rapid acylation of ampicillin.

**Table 3:** χ_1_ angles defining the rotamer conformations of Ser^130^ in CTX-M and other class A β-lactamase structures

Enzyme (chain)	Ser^130^ χ_1_ dihedral angle	PDB code	Reference
	*degrees*		
CTX-M-14 (A)	−141.43	1YLT	59
CTX-M-9 (A)	−142.38	2P74	23
CTX-M-9 (B)	−141.45	2P74	23
CTX-M-15 (A)	−137.93	4HBT	60
CTX-M-15/avibactam (A)	−146.18	4HBU	60
CTX-M-16 (A)	−150.30	1YLW	59
CTX-M-27 (A)	−144.20	1YLP	59
CTX-M-96 (A)	−146.51	3ZNY	61
Toho-1 (CTX-M-44) (A)	−154.19	1IYS	38
CTX-M-9 S70G (A)	−142.18	3HRE	48
CTX-M-9 S70G (B)	−142.69	3HRE	48
CTX-M-9 S70G/CTX (A)	−47.69, −143.10	3HLW	48
CTX-M-9 S70G/CTX (B)	−51.39, −147.43	3HLW	48
CTX-M-9 S70G/PenG (A)	−143.85	3HUO	48
CTX-M-9 S70G/PenG (B)	−143.24	3HUO	48
CTX-M-14 S70G (A)	−69.67	4PM6	21
CTX-M-14 S70G/CTX (A)	−64.02	4PM5	21
Toho-1 E166A/CTX (A)	−155.30	1IYO	37
Toho-1 E166A/PenG (A)	−164.23	1IYQ	37
CTX-M-14 E166A/K234R (A-H)	−55.75, −52.54, −58.54, −59.41, −43.92, −56.31, −52.92, −51.73	This work	
CTX-M-14 E166A/K234R/AMP (A)	−63.64	This work	
CTX-M-14 E166A/K234R/CTX (A)	−49.29	This work	
SHV-1 (A)	−149.64	1SHV	62
TEM-1 (A)	−137.82	1XBP	63
TEM-32 (A)	−53.65	1LI0	13
PSE-4 (A)	−69.66, −153.85	1G68	25
PSE-4 R234K (A)	−142.79	1G6A	25

We suggest that there is an equilibrium between the χ_1_ ∼−150 and ∼−60° rotamers of Ser^130^ in both the free enzyme and enzyme-substrate complexes. By this view, in the WT CTX-M-14 enzyme with Lys^234^, the equilibrium favors the χ_1_ ∼−150° rotamer, and Ser^130^ is positioned to function in the proton shuttle. The presence of arginine in the K234R enzyme, however, shifts the equilibrium toward the ∼−60° rotamer, as suggested by the E166A/K234R apoenzyme structure, where all eight molecules in the asymmetric unit are in this rotamer form (Fig. S1 and Table 3). A further indication that arginine at position 234 influences the Ser^130^ rotamer conformation is provided by the structure of the class A β-lactamase PSE-4, which has Arg^234^ in the native enzyme (25, 46). In the PSE-4 apoenzyme structure, there is a single molecule in the asymmetric unit, but Ser^130^ occupies two rotamer conformations with χ_1_ values of −70 and −154°, suggesting that the rotamers are in equilibrium (25) (Table 3 and Fig. S3B). Interestingly, the structure of a PSE-4 R234K mutant reveals a single Ser^130^ rotamer with χ_1_ of −143° (Fig. S3C), suggesting that the presence of Lys^234^ shifts the equilibrium toward the χ_1_ ∼−150° rotamer, where Ser^130^ can function in the proton shuttle. Also of interest is that the WT PSE-4 enzyme exhibits high *k*_cat_ and *k*_cat_/*K_m_* values for the hydrolysis of penicillins, including ampicillin, but hydrolyzes cefotaxime very poorly, with a *k*_cat_ that is nearly 2,000-fold lower than that observed for ampicillin hydrolysis, consistent with our findings with the CTX-M K234R enzyme (25, 47).

There is also structural evidence suggesting that the substrate influences the equilibrium of the rotamers at Ser^130^. The structure of CTX-M-9 S70G with intact benzylpenicillin bound shows Ser^130^ in the χ_1_ −150° rotamer in both molecules in the asymmetric unit, with the hydroxyl positioned to function in the proton shuttle (48) (Table 3). In contrast, in the structure of CTX-M-9 S70G with cefotaxime bound, Ser^130^ occupies both the χ_1_ −150 and −60° rotamers, suggesting that the presence of cefotaxime may shift the equilibrium toward the χ_1_ −60° rotamer. Therefore, the presence of the K234R substitution shifts the equilibrium toward the inactive χ_1_ −60° rotamer and binding of cefotaxime and may further stabilize the inactive χ_1_ −60° rotamer.

Previous studies of the TEM-1 S130G substitution associated with clavulanic acid resistance showed that ampicillin hydrolysis was reduced 10-fold, but cephalosporin hydrolysis was reduced 1,000-fold (12). The fact that the Ser^130^ side chain was absent yet the enzyme retained activity was attributed to the presence of a water molecule occupying the position where the Ser^130^ Oγ normally resides. Therefore, it was hypothesized that the water substitutes for the Ser^130^ hydroxyl group in the proton shuttle to the β-lactam nitrogen leaving group (12). Here, the CTX-M S130A mutant hydrolyzes ampicillin at near WT *k*_cat_ values, suggesting that acylation occurs rapidly. However, modeling the S130A substitution into the acyl-enzyme structures of E166A/K234R with ampicillin and cefotaxime suggests that the presence of the C-β methyl group of alanine does not leave sufficient space for a water molecule. We cannot exclude the possibility, however, that the dynamics of the enzyme in solution may allow water to access the site and replace the hydroxyl group in the S130A mutant and donate a proton to the β-lactam nitrogen to facilitate acylation.

It is important to note that cefotaxime has a good leaving group at the C3 position that is ejected during or subsequent to acylation (Figs. 2*C* and 3). Indeed, the E166A/K234R structure with cefotaxime shows a methylene group at C3, indicating that the R2 group has been eliminated (Fig. S2B). This raises the question of whether protonation of the N5 nitrogen is kinetically important for cefotaxime hydrolysis because a deprotonated N5 is required for elimination of the R2 group (Fig. 2*C*). It has been shown that elimination of the R2 group is not coincident with breakage of the β-lactam amide bond, and therefore protonation may occur before elimination, as has been suggested (49). Further, we found that cephalosporins with different leaving groups or with no leaving group (ceftizoxime) were similarly affected by the K234R substitution, suggesting that the substitution is not acting through changes in the rate of elimination of the leaving group (Table S1). Nevertheless, alternate hypotheses could explain the observed effect. For example, the role of the lysine at residue 234 could be to provide a more positive environment than arginine, favoring stabilization of the negative partial charges formed in the transition state in the case of cefotaxime but not ampicillin. This hypothesis is not mutually exclusive with altering protonation efficiency of the β-lactam nitrogen and may also contribute to the observed effects.

The K234R substitution has been identified in SHV variant β-lactamases in drug-resistant clinical isolates, where it increases the IC_50_ for clavulanic acid inhibition (15, 19, 27). Further, molecular dynamics simulations of the K234R substitution in SHV showed that the substitution favors the shifting of the Ser^130^ side chain to the χ_1_ −60° rotamer (15). Because clavulanic acid is known to covalently cross-link Ser^70^ and Ser^130^ in the inhibition mechanism, it was proposed that the shifting of the Ser^130^ side chain to the altered χ_1_ −60° conformation reduces clavulanic acid binding and the inhibitor potency (15). Here, we provide X-ray crystallographic evidence that the K234R substitution results in a shift of the Ser^130^ conformation to favor the χ_1_ −60° rotamer. Further, we show that this shift in conformation is also associated with an increase in the *K_i_* for clavulanic acid inhibition of CTX-M β-lactamase.

A number of amino acid substitutions in the TEM-1 and SHV-1 enzymes have been associated with clavulanic acid resistance (11). The S130G substitution occurs in both TEM and SHV variants and is associated with reduced inhibitor potency. It has been proposed that the removal of the hydroxyl at position 130 reduces potency because cross-linking of clavulanic acid between Ser^70^ and Ser^130^ cannot occur (11, 12, 14). The M69I substitution in TEM-32 has also been associated with reduced clavulanic acid potency (11, 13). Interestingly, the structure of TEM-32 shows that the Ser^130^ side chain is shifted from χ_1_ −140° in WT TEM-1 to χ_1_ −54° on introduction of the M69I mutation (13) (Table 3). Thus, the M69I substitution results in a Ser^130^ conformational change very similar to that observed for the K234R CTX-M enzyme structures reported here. These observations support the idea that the Ser^130^ conformational change is the cause of decreased clavulanic acid potency (13).

Finally, it has previously been suggested that clavulanic acid should be combined with a cephalosporin rather than amoxicillin to avoid inactivation of the β-lactam drug due to the induction of AmpC β-lactamases by clavulanic acid in many *Enterobacteriaceae* species (50, 51). Our results suggest that the combination of clavulanic acid with a cephalosporin may be useful for a different reason *i.e.,* Ser^130^ and Lys^234^ variants that lead to clavulanic acid resistance are poor catalysts for oxyimino-cephalosporin hydrolysis, and thus the bacteria harboring these mutants would not be protected from cell death induced by the cephalosporin. In contrast, Ser^130^ and Lys^234^ variants readily hydrolyze ampicillin, an antibiotic that only differs from amoxicillin by lacking one hydroxyl group on the benzene ring. Therefore, the evolution of resistance due to these mutations would be blocked by the combination of clavulanic acid with an oxyimino-cephalosporin but not a penicillin.

## Experimental procedures

### Strains and plasmids

Plasmids pET28a-CTX-M-14, pET28a-CTX-M-14^E166A^, and pET24a-TEM-1 used in this study were described previously (36, 52, 53). The *E. coli* strain XL1-Blue [*recA1, endA1*, *gyrA96, thi-1, hsdR17*, *supE44*, *relA1, lac*, [F9 proAB lacIq lacZΔM15, Tn10 (tetr)]] (Stratagene, Inc., La Jolla, CA, USA) was used as the host for the construction of CTX-M-14 and TEM-1 variants. The *E. coli* strain BL21(DE3) (*fhuA2 [lon] omp Tgal (*λ*DE3) [dcm]* Δ*hsdS* λ *DE3*=λ *sBamHIo* Δ*EcoRI-B int::(lacI::PlacUV5::T7gene1) i21* Δ*nin5*) was used as the host for protein expression.

### Site-directed mutagenesis and K234X library construction

pET28a-CTX-M-14, pET28a-CTX-M-14^E166A^ and pET24a-TEM-1 were used as templates for PCR, respectively. Amino acid substitution was introduced into position Lys^234^ using the following primers: K234R-CTXM-M-14 (5′-GGACTGTGGGTGATCGTACCGGCAGCGGCGACTAC-3′ and K234R-TEM-1 (5′-CTGGTTTATTGCTGATCGGTCTGGAGCCGGTGAG-3′). DNA amplifications were performed using Phusion® High-Fidelity DNA Polymerase (New England BioLabs, Ipswich, MA, USA), following the manufacturer's guidelines. DNA sequences of the plasmids with the corresponding K234R mutation were confirmed by Eurofins (Luxembourg, KY, USA).

The K234X randomized codon library was constructed by first inserting a XhoI restriction site at position Lys^234^ and simultaneously deleting a base pair from the coding sequence to create a frameshift mutation. This mutant was constructed by oligonucleotide-directed mutagenesis using the QuikChange method to create plasmid pTP715. This plasmid then served as template for randomization of position 234 by oligo-directed mutagenesis. The added XhoI site is unique on the pTP123:CTX-M-14 plasmid, enabling efficient linearization and removal of unsuccessful mutagenesis reactions during library construction. Likewise, the frameshift mutation yields truncated CTX-M-14 proteins with expected loss of function, allowing further removal of template gene during subsequent functional screens.

The K234X library was constructed using a single primer and site-directed mutagenesis using New England Biolabs Phusion High Fidelity polymerase (catalog no. M0530L) and the manufacturer's recommended protocol. Position 234 was randomized to NNS, where N represents a mixture of all four nucleotides and S represents a mixture of G and C. After the PCR, 20 units of DpnI (New England Biolabs, catalog no. R0176L) and 20 units of XhoI (New England Biolabs catalog no. R0146L) were added directly to the PCR product and incubated for ∼16 h at 37 °C. The PCRs were purified using Qiagen Minelute columns and eluted using 10 μl of 10 mm Tris-Cl, pH 8.5. Afterward, 5 μl of the reaction was added to 50 μl of *E. coli* XLI-Blue electro-competent cells and electroporated at 200 ohms, 1.7 mV, and 15 microfarads. The cells were recovered in 500 ml of prewarmed (37 °C) SOC medium and incubated at 37 °C for 1 h, shaking at 180 rpm. The recovered cells were then spread on 100 ml of Luria–Bertani (LB) agar plates with 12.5 μg/ml chloramphenicol (CMP) and allowed to grow for ∼20 h at 37 °C. Afterward, the library transformants were pooled using 1.5 ml of LB medium with 12.5 μg/ml CMP, and pooled library plasmid DNA was extracted using Qiagen Spin Miniprep columns (catalog no. 27106). Greater than 1,000 colonies were pooled to constitute the K234X library.

### K234X library antibiotic selection

The K234X library selection experiment was performed in *E. coli* XL1-Blue grown in Mueller–Hinton broth (BD 212322) with 10 mg/ml MgCl_2_, 20 mg/ml CaCl_2_, and 12.5 μg/ml CMP, in 1.2-ml round-bottom 96-well plates (catalog no. Axygen P-DW-12-HC-S) and shaking at 180 rpm at 37 °C for 20 h. In short, ∼50 ng of library DNA was transformed into *E. coli* XLI-Blue electrocompetent cells at 200 ohms, 1.7 mV, and 15 microfarads. The cells were recovered in 500 μl of prewarmed (37 °C) Mueller–Hinton broth at 37 °C for 1 h with shaking at 180 rpm. The library cells were diluted 10-fold into Mueller–Hinton broth with 10 mg/ml MgCl_2_, 20 mg/ml CaCl_2_, and 12.5 μg/ml CMP, in 1.2-ml round-bottom 96-well plates and selected against 0, 2, and 32 μg/ml ampicillin and 0, 0.0625, and 0.25 μg/ml cefotaxime. The cultures were allowed to grow for 20 h shaking at 180 rpm at 37 °C.

### PCR amplification and NGS deep sequencing

Plasmid DNA from pooled colonies after antibiotic selection, or from the naive library control, was used as template DNA for PCR amplification. A 160-bp region containing the randomized position 234 was amplified for each selected K234X library. PCR primers also included 8-bp barcodes and ligation adapters for downstream NGS sequencing. PCR products were purified from a 1.5% agarose gel using QIAquick DNA extraction kit (Qiagen). The DNA of each purified product was determined using a Nanoquant plate (TECAN). PCR products from each selected library and the naive control were pooled into a single tube, and Illumina deep sequencing was performed by Novogene Co., Ltd. (read length 150 bp).

### Computational processing of NGS data

Analysis of NGS deep-sequencing FASTQ files was performed using a custom Python 3.0 script. The selection condition of each read was determined based on the 8-bp barcode, and the codon at the randomized position 234 was identified based on the 6 bp upstream and 6 bp downstream of the codon of interest. This yielded 5.3 × 10^6^ total reads, with an average of 1 × 10^6^ reads for each of the five selection conditions (including the naive library). The number of times each amino acid occurred at position 234 in each condition was counted to give the totals used in the text. The sequence logo representing these results was created using the seqlogo function in MATLAB. The amino acid distribution for each antibiotic selection condition was normalized to that of the naive library to account for biases prior to antibiotic selection.

### Protein expression and purification

CTX-M-14 WT and its variants were expressed with an N-terminal His_6_ tag and purified from *E. coli* BL21(DE3) strains as described previously, with certain modifications (36). *E. coli* cells were cultured in LB medium at 37 °C until OD reached 1.0–1.2. Protein overexpression was induced by 0.2 mm isopropyl β-d-1-thiogalactopyranoside for 20 h at 23 °C. The next day, cells were harvested by low-speed centrifugation and resuspended in buffer A (25 mm sodium phosphate (pH 7.4), 300 mm NaCl, and Xpert protease inhibitor mixture (GenDEPOT, Katy, TX, USA) supplemented with 20 mm imidazole. Sonication was used to disrupt cells, and cell debris was removed by centrifugation at 80,000 × *g* for 15 min. Supernatant was loaded onto a column packed with Talon resin (Takara Bio USA, Inc., Mountain View, CA, USA), and the flow-through was collected. This step was repeated twice to increase protein binding. After washing, protein was eluted by adding buffer A supplemented with 40, 60, and 80 mm imidazole, respectively. Protein fractions were then combined, concentrated, and buffer-exchanged with buffer A using an Amicon® Ultra-15 centrifugal filter unit (MilliporeSigma, Burlington, MA, USA). The N-terminal His tag of the purified protein was cleaved by treatment with tobacco etch virus protease overnight at 4 °C. Tag-free CTX-M-14 proteins were obtained after incubating with nickel-Sepharose 6 Fast Flow resin (GE Healthcare Life Sciences) for 1 h at 4 °C. Protein purity and His tag cleavage were visualized and confirmed by SDS-PAGE followed by Coomassie Brilliant Blue staining.

### Enzyme kinetics

Michaelis–Menten steady-state kinetic parameters of CTX-M-14 β-lactamases were determined on various β-lactam substrates. Wavelength and extinction coefficients used for detection were as follows: ampicillin, 235 nm, Δε = 900 m^−1^ cm^−1^; cephalothin, 262 nm, Δε = 7,660 m^−1^ cm^−1^; cefotaxime, 264 nm, Δε = 7,250 m^−1^ cm^−1^; nitrocefin, 482 nm, Δε = 15,000 m^−1^ cm^−1^; cefpodoxime, 235 nm, Δε = 1,023 m^−1^ cm^−1^; ceftriaxone, 241 nm, Δε = 2,046 m^−1^ cm^−1^. Enzymatic reactions were performed at 25 °C in buffer containing 50 mm sodium phosphate (pH 7.0) and 1 μg/ml BSA. Substrate hydrolysis was monitored by Beckman Coulter spectrophotometer model DU 800 (Beckman Coulter, Brea, CA, USA). Initial hydrolysis velocities of substrate were plotted as a function of concentrations and fitted into a Michaelis–Menten equation using GraphPad Prism 5 (GraphPad Software, San Diego, CA, USA) to obtain *K_m_* and *k*_cat_ values. The *k*_cat_/*K_m_* values for the enzymes where *k*_cat_ and *K_m_* could not be determined independently were estimated using the equation, ν = (*k*_cat_/*K_m_*)[*E*][*S*], where [*S*] ≪ *K_m_* (54).

Single-turnover kinetics experiments were used to determine the acylation rate for the CTX-M-14 WT and K234R enzymes. Kinetic measurements were carried out at 25 °C using a using a KinTek stopped-flow instrument. Protein concentrations used in this experiment exceed the substrate concentrations. To prepare sample injection, 1 ml of increasing concentrations of CTX-M-14 proteins and 1 ml of 2.5–5 μm cefotaxime were loaded into two separate drive syringes. 5 μm cefotaxime was used for single-turnover experiments with WT CTX-M-14, whereas 2.5 μm cefotaxime was used in experiments with the K234R enzyme. Data collection times were adjusted empirically. Absorbance at 264 nm was monitored after samples in two syringes were mixed. For each selected time period, 1,000 data points were collected, and at least three kinetic traces were obtained to ensure high-quality data. Kinetic data with various protein concentrations were fitted to a single-exponential function, $A_{t}=A^{\left(-k_{t}\right)}+C$, to determine *k*_obs_, which was then used to determine *k_2_* by fitting *k*_obs_ as a function of enzyme concentration to a hyperbola, as has been reported previously for serine β-lactamases and serine proteases (33, 34, 35). The deacylation rate constant, *k_3_*, was calculated from the experimental values determined for *k*_cat_ and *k*_2_, using the equation, *k*_cat_ = *k*_2_ × *k*_3_/(*k*_2_ + *k*_3_). Solving the equation for *k*_cat_ in terms of *k*_3_ gives *k*_3_ = (*k*_2_ × *k*_cat_)/(*k*_cat_ − *k*_2_). The error in the calculated value of *k*_3_ was determined by propagating the errors in *k*_2_ and *k*_cat_ through the equation by adding the absolute errors for the sum (difference) in the denominator to obtain the absolute and the percentage error in *k*_cat_ − *k*_2_. The percentage error of the numerator, (*k*_cat_ × *k*_2_), was calculated by adding the percentage error for *k*_cat_ and *k*_2_, and the final percentage and absolute errors on *k*_3_ were then obtained by adding the percentage error of the numerator and denominator to yield the percentage error in *k*_3_.

The *K_i_* values for clavulanic acid inhibition of CTX-M-14 WT and variants were determined by mixing the enzymes with increasing concentrations of clavulanic acid in buffer containing 50 mm sodium phosphate (pH 7.0) and 1 μg/ml BSA and incubating at room temperature for 20 min. Enzyme activity was evaluated by monitoring the initial velocity of hydrolysis of 100 μm ampicillin using Beckman Coulter spectrophotometer model DU 800. The percentage activity was calculated by dividing the initial hydrolysis velocity for ampicillin in the presence of clavulanic acid by the velocity in the absence of clavulanic acid and plotted as a function of clavulanic acid concentration. The *K_i_* values were obtained by fitting the data into Morrison equation using GraphPad Prism 5 (55).

### X-ray crystallography

Crystal screening was performed using commercially available crystal screens PEGs and PACT from Qiagen (Valencia, CA, USA) and the hanging-drop vapor diffusion method, which was set up by an in-house TTP LabTech Mosquito instrument (TTP Labtech Ltd., Melbourn, UK). CTX-M-14^K234R/E166A^ was concentrated to a final concentration of 24 mg/ml in buffer 10 mm Tris-HCl (pH 8.0) and 100 mm NaCl. CTX-M-14^K234R/E166A^ formed crystals in multiple conditions. CTM-M-14^K234R/E166A^ crystallized in condition (0.2 m CaCl, 0.1 m Tris-Cl, pH 8.0, 20% (w/v) PEG 6000) was used. To obtain CTX-M-14^K234R/E166A^ and substrate complexes, CTX-M-14^K234R/E166A^ crystals (0.1 m Tris-HCl, pH 8.5, and 25% (w/v) PEG 3000) and CTX-M-14^K234R/E166A^ crystals (0.2 m lithium chloride, 0.1 m Tris-HCl, pH 8, 20% (w/v) and PEG 6000) were soaked with 100 mm ampicillin and 50 mm cefotaxime, respectively, for 1 h at room temperature. Crystallization conditions supplemented with 25% glycerol were used as the cryoprotectant in individual cases.

Diffraction data were collected at the Berkeley Center for Structural Biology using the Advanced Light Source synchrotron beam line. Reflection data were indexed, integrated, and scaled using the HKL2000, iMosflm, and the CCP4i Suite (56). Molecular replacement was performed using CTX-M-14 (PDB entry 1YLT) as a structural model. Structures were further refined for several rounds with Phenix.refine and Coot density fitting (57, 58). The refined coordinates along with the structure factors have been deposited in the Protein Data Bank (entries 7K2X, 7K2Y, and 7K2W). The data collection and refinement statistics for all structures are listed in Table S2. The UCSF Chimera program was used to construct structure figures.

## Data availability

Coordinates and structure factors have been deposited in the Protein Data Bank under accession codes 7K2X, 7K2Y, and 7K2W. All relevant data associated with the paper are available upon request from the corresponding author.
