# Supplementary material for: A drug-resistant β-lactamase variant changes the conformation of its active-site proton shuttle to alter substrate specificity and inhibitor potency
Source: J Biol Chem. 2025 Jan 13;295(52):18239–55. doi: 10.1074/jbc.RA120.016103 (PMC11843585; doi:10.1074/jbc.RA120.016103)
Supplement: Supplementary file 1 [file mmc1.docx]

**Supporting Information**

A Drug-Resistant Variant Changes the Conformation of the β-lactamase Active Site Proton Shuttle to Alter Substrate Specificity and Inhibitor Potency

Victoria Soeung^1&^, Shuo Lu^1&^, Liya Hu^2^, Allison Judge^2^, Banumathi Sankaran^4^, B.V. Venkataram Prasad^2,3^ and Timothy Palzkill^1,2,3*^

**Table S1**. Enzyme kinetic parameters for wild-type CTX-M-14 and K234R β-lactamases with cephalosporin substrates.

**Table S2.** X-ray crystallography data collection and refinement statistics for CTX-M-14 K234R mutant enzymes.

**Figure S1.** Active site region of the CTX-M E166A/K234R apo structure.

**Figure S2.** F_o_-F_c_ omit maps showing electron density for acyl-enzyme intermediates with CTX-M E166A/K234R.

**Figure S3**. Active site region of the CTX-M-9 and PSE-4 enzymes.

**Table S1**. Enzyme kinetic parameters for wild-type CTX-M-14 and K234R β-lactamases with cephalosporin substrates. Ratio represents the ratio of the wild-type *k*_cat_/K_M_ to the *k*_cat_/K_M_ of the K234R substitution.

**Table S2.** X-ray crystallography data collection and refinement statistics for CTX-M-14 K234R mutant enzymes.

| Data collection | CTX-M-14 E166A/K234R | CTX-M-14 E166A/K234R/AMP | CTX-M-14 E166A/K234R/CTX |
| --- | --- | --- | --- |
| space group | P 32 | P 32 2 1 | P 32 2 1 |
| *a*, *b*, *c* (Å) | 83.37, 83.37, 232.09 | 41.63, 41.63, 231.47 | 41.65, 41.65, 232.31 |
| α,β,γ (°) | 90.00, 90.00, 120.00 | 90.00, 90.00, 120.00 | 90.00, 90.00, 120.00 |
| resolution range (Å) | 45.2 – 1.8 (1.82 - 1.80) | 30.6 - 1.62 (1.68 - 1.62) | 34.45 - 1.40 (1.45 - 1.4) |
| *R*_merge_ (%) | 8.7 (62.1) | 7.4 (25.8) | 3 (7.2) |
| *I*/sigma | 20.6 (3.3) | 18.0 (7.5) | 35.1 (15.7) |
| completeness (%) | 99.7 (99.8) | 98.4 (97.6) | 97.6 (99.9) |
| Wilson B-factor (Å^2^) | 18.31 | 17.46 | 9.97 |
| molecules per asymmetric unit | 8 | 1 | 1 |
| no. of unique reflections | 166642 (16637) | 30488 (2957) | 46582 (4684) |
| *R*_work_/*R*_free_ (%) | 18.1/22.4 | 18.8/22.6 | 19.9 / 24.4 |
| no. of protein atoms | 15500 | 1945 | 1936 |
| average B-factor (Å^2^) | 21.73 | 29.48 | 20.67 |
| protein | 20.83 | 28.30 | 18.23 |
| solvent | 30.65 | 39.15 | 32.88 |
| ligand | - | 29.29 | 23.83 |
| RMS deviations |  |  |  |
| bond length (Å) | 0.008 | 0.006 | 0.004 |
| bond angles (deg) | 0.9 | 0.82 | 0.82 |
| PDB codes | 7K2X | 7K2Y | 7K2W |

**Figure S1.** Active site region of the CTX-M E166A/K234R apo structure. The structure was determined with 8 molecules in the asymmetric unit. A structural alignment of the 8 chains (A-H) is shown for residues Ser70, Lys73, Ser130 and Arg234. Note that in all eight molecules the χ_1_ dihedral angle is ~55°, which is also observed for the E166A/K234R acyl-enzyme structures in complex with ampicillin and cefotaxime.


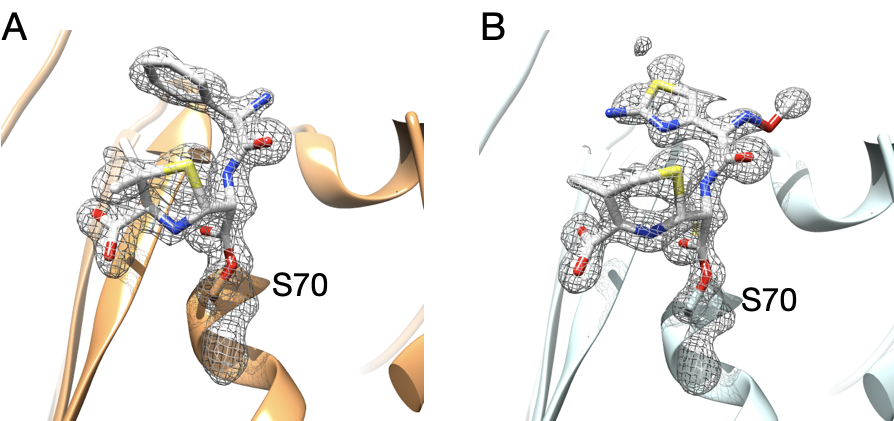


**Figure S2.** F_o_-F_c_ omit maps showing electron density for acyl-enzyme intermediates with CTX-M E166A/K234R. **A**. Omit map contoured at 3σ for the acyl-enzyme with ampicillin. Continuous electron density is present between Ser70 Oγ and ampicillin. Ampicillin carbons are shown in white, nitrogens in blue, and oxygens in red. Carbons in the E166A/K234R enzyme are shown in tan. **B**. Omit map contoured at 3σ for the acyl-enzyme with cefotaxime. Continuous electron density is present between Ser70 Oγ and cefotaxime. Cefotaxime carbons are shown in white and carbons in the E166A/K234R enzyme are shown in light blue.

**Figure S3**. Active site region of the CTX-M-9 and PSE-4 enzymes. **A**. Structure of CTX-M-9 S70G complex with penicillin G (3HUO). Hydrogen bond network of key amino acids of the proton shuttle including Lys73, Ser130 and K234. Note Ser130 is in the χ_1_ -143° rotamer conformation. **B**. Structure of the PSE-4 active site region showing the hydrogen bond network of the proton shuttle residues Lys73, Ser130 and R234 (1G68). Note Ser130 is in two rotamer conformations, χ_1_ -70° and -154°. **C**. Structure of the PSE-4 R234K active site region of proton shuttle residues (1G6A). Note Ser130 is in the χ_1_ -143° rotamer conformation.
